# The origin of nausea in migraine–A PET study

**DOI:** 10.1186/1129-2377-15-84

**Published:** 2014-12-03

**Authors:** Farooq H Maniyar, Till Sprenger, Christoph Schankin, Peter J Goadsby

**Affiliations:** 1 Headache Group – Basic & Clinical Neurosciences, King’s College London, London, UK; 2 Department of Neurology, University of California, San Francisco, San Francisco, CA, USA; 3 Basildon and Thurrock University Hospital, Nethermayne, Basildon SS16 5NL, UK; 4 The Royal London Hospital, Whitechapel, London E1 1BB, UK; 5 Department of Neurology and Division of Neuroradiology, University Hospital Basel, Petersgraben 4, 4031 Basel, Switzerland; 6 Department of Neurology, University of Munich Hospital – Großhadern, Marchioninistr. 15, D-81377 Munich, Germany; 7 NIHR-Wellcome Trust King’s Clinical Research Facility, King’s College Hospital, London SE5 9PJ, UK

**Keywords:** Nausea, Premonitory, PET, NTS, Trigeminal

## Abstract

**Background:**

Nausea is a common and disabling symptom of migraine. The origin of nausea is not well understood although functional connections between trigeminal neurons and the nucleus tractus solitarius may explain occurrence of nausea with pain. However, nausea occurs as a premonitory symptom in about a quarter of patients, suggesting that a primary brain alteration unrelated to the experience of pain may be the reason for nausea.

**Methods:**

We performed positron emission tomography scans with H_2_^15^O PET in premonitory phase of nitroglycerin-induced migraine and compared patients with and without nausea.

**Results:**

The results showed activation in rostral dorsal medulla and periaqueductal grey (PAG) in the nausea group, which was absent in the no nausea group. The rostral dorsal medullary area included the nucleus tractus solitarius, dorsal motor nucleus of the vagus nerve and the nucleus ambiguus, all of which are thought to be involved in brain circuits mediating nausea.

**Conclusions:**

The results demonstrate that nausea can occur as a premonitory symptom in migraine, independent of pain and trigeminal activation. This is associated with activation of brain structures known to be involved in nausea. We conclude that nausea is a centrally driven symptom in migraine.

## Background

Nausea is a common and disabling symptom of migraine, which is listed in the International Classification of Headache Disorders-III-beta (ICHD-III-beta) as a key symptom of an attack [1]. The neurobiology of nausea in migraine is not well understood. Nausea is often associated with the pain in migraine and in this regard, connections between trigeminal neurons and nucleus tractus solitarius (NTS) are thought to explain the occurrence of nausea with headache [2]. However, nausea can already be present in the premonitory phase, before the appearance of headache.

In a previous study, nausea in the premonitory phase correctly predicted headache in a quarter of migraineurs [3]. The presence of nausea before pain suggests it can occur independent of pain. Premonitory symptoms represent the earliest clinical change in migraineurs. Premonitory symptoms, such as tiredness, difficulty in concentration, mood changes, yawning and cravings, suggest the origin of the problem is likely to be within the brain, and therefore, nausea could also be a predominantly centrally driven symptom. We hypothesized that central brain structures involved in nausea and emesis, in particular, the NTS, is activated in migraine patients who experience nausea in the absence of pain in the premonitory phase. We conducted positron emission tomography (PET) scans as a marker of neuronal activity in the premonitory phase of migraine to be able to compare patients with and without nausea.

## Methods

Patients included in this study were screened for an over-arching investigation of the premonitory phase of GTN-induced migraine [4]. After completion of PET scans, a pre-planned sub-group analysis was conducted between patients with and without nausea in the premonitory phase.

### Recruitment

We conducted telephone interviews, after advertisements in local media, to select patients who met the inclusion criteria: age 18–65 years, migraine without aura [5], less than fifteen days of headache a month, premonitory symptoms before headache [3], no major medical conditions and not on preventive drugs for migraine or any other regular medications, which could confound the study. We excluded patients with migraine aura to prevent confusion with premonitory symptoms, as both usually occur before headache. The study was approved by the UCSF Committee on Human Research and the Radiation Safety Committee. Written informed consent was obtained from all patients before study inclusion.

### Screening

We initially screened one-hundred and twenty-five patients. Twenty-seven satisfied the inclusion criteria and agreed to participate. The most common reasons for non-inclusion were: headache for more than fifteen days a month (*n* = 45), confounding medications (*n* = 29), migraine aura (*n* = 12), and patients unwilling due to radiation risk (*n* = 8); less common reasons for exclusion are included in a table (Table 1).

**Table 1:** Miscellaneous reasons for non-inclusion after telephonic screening

	**Reasons for non-inclusion**	***n***
1	History not compatible with ICHD defined migraine	6
2	No premonitory symptoms before headache during spontaneous attacks	5
3	Difficulty contacting patients after initial eligibility	4
4	Incomplete screenings	4
5	Outside age-range	2
6	Patient refusals due to GTN risk	3
7	Patient refusals due to – time factors, inadequate compensation, lack of interest or unwilling to get induced migraine	7
	Total	31

### GTN triggering

These twenty-seven patients were invited for the first visit during which we infused intravenous GTN 0.5 micrograms × kg^-1^ × minute^-1^ over 20 minutes to select patients who responded with premonitory symptoms followed by a delayed headache resembling their migraine headache, hereafter referred to as migraine headache [6]. Details about headache and associated symptoms were asked initially every five minutes and then less frequently (Table 2). Blood pressure, oxygen saturation and pulse rate were recorded initially every five minutes and then less frequently. Of these twenty-seven patients, eighteen had migraine headache. Out of these eighteen, thirteen patients had premonitory symptoms before migraine headache. These thirteen patients were invited for PET scans at least seven days after the first GTN infusion.

**Table 2:** Recording of symptoms after triggering with GTN

**A**	**B**
Time points after GTN infusion initiation	Symptoms queried at each time point
-5	Headache details – side, site, type
0	Nausea
5	Photophobia
10	Phonophobia
15	Tiredness
20	Neck Stiffness
25	Yawning
30	Mood Changes
35	Urination
40	Cravings
50	Thirst

Recording of symptoms was continued at 10–15 minute intervals until all scans were complete.

### PET scanning

All patients had been pain free at least 72 hours before the PET scans. The procedure for GTN infusion and recording of symptoms was repeated as on the first occasion. We performed PET scans with the GE Discovery VCT PET/CT system (Waukesha, WI, United States) in three-dimensional mode with septa retracted. All subjects were instructed to keep their eyes closed during the scans. The subjects were positioned in the PET scanner, and their head immobilized with standard immobilization straps and a low dose CT scan was performed for attenuation correction. CT scans for attenuation correction were repeated when patients exited the scanner for relaxation between conditions. An antecubital vein cannula was used to administer the tracer, 370 MBq of H_2_^15^O, which was repeated before each scan. The activity was infused into subjects over twenty seconds at a rate of 10 ml/min. The interval between scans was at least ten minutes allowing an interval of five half-lives of H_2_^15^O (t_1/2_ = 122 seconds). The PET data were acquired dynamically and summed for one 90-second frame beginning five seconds before the peak of the head curve.

### Scans

Each patient had scans in three conditions – baseline (pain free), premonitory phase (pain free) and migraine headache. We could not randomize the order of the scans since the premonitory and migraine headache phases were triggered by the GTN infusion sequentially. We planned to do three scans in each condition. However, the number of scans in the premonitory phase depended on how soon the migraine headache developed. Soon after the initiation of GTN, patients had a mild headache that lasted for a mean of 23 ± 7 minutes (range 11–32). Premonitory scans were performed after the GTN headache had completely subsided, premonitory symptoms were present and the migraine headache had not appeared. The mean time for the first premonitory scan after initiation of GTN infusion was 56 ± 16 minutes (range 38–80). Migraine scans were performed when the migraine headache (delayed headache) was moderate or severe. The mean time for the first migraine scan after initiation of GTN infusion was 125 ± 41 minutes (range 89–225). Subsequent scans in the premonitory and migraine phases were conducted at approximately 10 minute intervals. Out of thirteen, ten patients had at least one scan during the premonitory period when no pain was present. Therefore, only these ten patients’ scans were used for the final analysis.

All the ten patients had three scans each during baseline. In the premonitory phase, five patients had two scans each, three patients had one scan each; one patient had three and one patient had four scans. In the migraine headache phase, five patients had three scans each, two patients had two, one patient had one, one patient had four and one patient had five scans. Images were reconstructed by 3D iterative reconstruction (3DIR) provided by the manufacturer into 47 image planes (separation 3.27 mm) and into a 128 × 128 pixel image matrix (pixel size 2.1 × 2.1 mm^2^).

### Statistical analysis

SPM2 (*http://www.fil.ion.ucl.ac.uk/spm*) was used for data preprocessing and statistical analysis [7]. Images were realigned with the first as reference and stereotactically normalized into MNI space. The normalized images were smoothed with a Gaussian filter of 8 mm full width at half-maximum. Statistical parametric maps were derived with pre-specified contrasts, comparing rCBF (Regional Cerebral Blood Fow) during states of interest. Using the brainstem as the region of interest (ROI), we specifically looked for differences in the two groups: nausea and no nausea in the premonitory phase, in areas known to be involved in nausea like the rostral dorsal medulla containing the NTS, dorsal motor nucleus of the vagus nerve, nucleus ambiguus and area postrema [8-10]. Since there are no known MNI coordinates representing these areas, we used the human atlas by DeArmond [11] to approximate the location of activations. We initially carried out a paired *t*-test to study activations in the premonitory phase > baseline, in the nausea group, and then carried out the same procedure separately for the no-nausea group. Finally, we used a two sample *t*-test to study activations in the premonitory phase > baseline, in the nausea group > no nausea group. As we had a strong hypothesis regarding activations in the lower brainstem in the nuclei noted above, we initially looked at the results with a threshold *p* < 0.05, uncorrected for multiple comparisons. Activated clusters in the above-mentioned areas were selected. We then carried out a small volume correction (SVC) for multiple comparisons within a sphere of radius 5 mm, centered at co-ordinates representing maxima within this cluster. *P* < 0.05 after false discovery rate (FDR) correction for multiple comparisons was deemed significant.

## Results

The premonitory symptoms in the two groups during the scanning session are listed in Table 3.

**Table 3:** Premonitory symptoms in GTN-induced migraine during the scanning session

**Nausea group**	
Patient number	Premonitory symptoms
1	Nausea, photophobia, thirst
2	Nausea, photophobia, tiredness, mood changes
3	Nausea, tiredness, neck stiffness, urination, thirst, dry mouth
**No nausea group**	
Patient number	Premonitory symptoms
1	Tiredness, neck stiffness, yawning
2	Photophobia, tiredness, neck stiffness, mood changes
3	Urination, thirst, neck stiffness
4	Yawning, urination, thirst
5	Tiredness
6	Photophobia, neck stiffness, tiredness
7	Photophobia, neck stiffness, thirst

Three patients had nausea and seven did not have nausea in the premonitory phase during the scanning session. The age range was 19–47 years (32 ± 10, mean ± SD). Patients in the two groups did not differ in age (unpaired tailed *t*-test, *p* = 0.6). There were two males in the nausea group and one male in the no nausea group.

**Figure 1:**
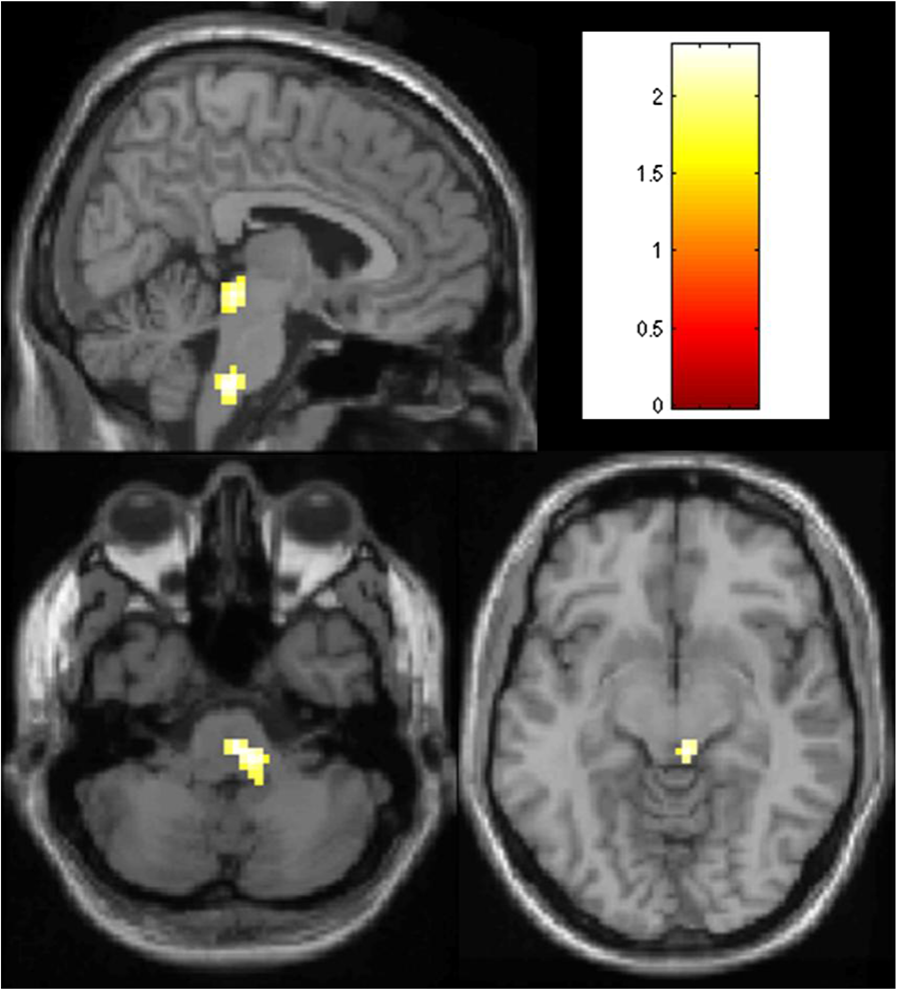
**Areas of increased rCBF in the premonitory phase of migraine in patients with nausea > patients without nausea.** Areas of increased rCBF in the premonitory phase > baseline are depicted in patients with nausea > patients without nausea. The results are superimposed on an anatomical reference derived from a representative T1-weighted MRI of one of the patients in this study. The colour bar indicates the colour coding for the Z scores. Images are displayed in radiological convention i.e. left side of image is right side of brain. A threshold of *p* < 0.01, uncorrected, was used for display purposes.

Comparing the premonitory scans > baseline scans in the nausea group with an uncorrected p value threshold of *p* < 0.05, we found brainstem activations in the rostral dorsal medulla and the PAG. These results survived SVC for multiple comparisons within a sphere of radius 5 mm centered at co-ordinates representing maxima within that cluster. Comparing the premonitory scans > baseline scans in the no-nausea group with an uncorrected threshold of *p* < .05, we did not find activations in the brainstem. Finally, comparing the premonitory scans > baseline scans, in the nausea group > no-nausea group with an uncorrected threshold of *p* < 0.05, we again found activations in the dorsal rostral medulla and the PAG, which survived SVC for multiple comparisons within a sphere radius of 5 mm centered at co-ordinates representing maxima within that cluster (Figure 1 and Table 4). Referring to the atlas by DeArmond [11], the activations in the rostral medulla coincided with the NTS, dorsal motor nucleus of the vagus nerve and nucleus ambiguus.

**Table 4:** MNI co-ordinates and statistical values of a priori areas with increased rCBF in the premonitory phase as compared to baseline in patients with nausea more than patients without nausea

**Brain regions**	**MNI coordinates**			**Z score**	***P *** **value**
	**X**	**Y**	**Z**		
Left rostral dorsal medulla	-6	-30	-45	2.23	< 0.03*
Left PAG	-6	-30	-9	2.18	< 0.04*

The co-ordinates represent the maxima within an activation cluster.

*after SVC for multiple comparisons using false detection rate (FDR) within a sphere of radius 5 mm centered at the given co-ordinates.

## Discussion

Our findings link nausea in the premonitory phase of migraine to activation of central structures in the rostral dorsal medulla and the PAG. The activation in the rostral dorsal medulla included the NTS, dorsal motor nucleus of vagus and nucleus ambiguus, all of which are known to be important central mediators of nausea. Primary activation of these nuclei exclusively in patients who experience nausea before the appearance of headache and trigeminal activation, demonstrates that nausea can be a centrally driven symptom in some patients.

Various brain areas are involved in nausea and emesis without there being a single ‘emesis center’. Areas in the dorsal rostral medulla, including all the areas activated in our study have been proposed to be particularly important. The NTS plays a coordinating role and receives information from vagal afferents and the area postrema [8-10]. The nucleus ambiguus is involved in the respiratory related components of vomiting [12]. The dorsal motor nucleus of the vagus nerve causes relaxation of the lower esophageal sphincter and the gastric fundus, both of which precede emesis [13]. The PAG modulates the function of the nucleus ambiguus [14]. The PAG can also be associated with sympathetic system activation seen during nausea and emesis [15,16]. Cardiovascular and respiratory regulation by the PAG is mediated through ascending projections to the dorso-medial hypothalamus and descending projections to the ventral medulla [17]. The NTS has afferent and efferent connections with the paraventricular nucleus of the hypothalamus suggesting a role in autonomic control [18]. The paraventricular and dorso-medial hypothalamic nuclei form the principal central afferent input to the area postrema [10]. Brain areas rostral to the nucleus ambiguus are not essential for vomiting, but may initiate vomiting under certain circumstances such as to cancer chemotherapy or psychogenic vomiting [19]. In this regard, electrical stimulation of the preoptic and medial nuclei of the hypothalamus, the nucleus anterior ventralis of the thalamus and limbic cortical areas, such as the amygdala and hippocampus, have been shown to induce vomiting [20]. When we compared premonitory scans of all patients (with and without nausea) against baseline, as we have reported [4], we found activations in the dorsal and lateral regions of the hypothalamus but not in anterior thalamus, hippocampus or amygdala. Therefore, considering the connections between the NTS and area postrema with the hypothalamus, the hypothalamus may play a direct or indirect role in nausea in the premonitory phase. The absence of hypothalamic activation in the nausea group could be due to low number of patients.

We cannot be certain that activation in the rostral caudal medulla itself causes nausea and vomiting or alternatively these areas are secondarily activated by other sub-cortical or cortical areas. However, the important finding is that the medullary areas are activated before the appearance of pain and hence do not represent a reaction to pain. It would therefore be reasonable to believe that nausea is centrally generated within the brain in the premonitory phase of migraine. Worsening of nausea during headache is likely to be due to the activation of functional connections between the trigeminovascular neurons and the NTS [2,21].

### Limitations

It needs to be emphasized that studying nausea in migraine is very difficult, because patients are lying immobilized in the scanner and nausea potentially carries the risk of vomiting with aspiration. No neuroimaging study has specifically addressed nausea in migraine to date. We used data where nausea was recorded, although it was not the main outcome of the principal study. The number of patients in this pilot study was small, especially in the nausea group and group sizes are not balanced. However, each patient had three scans in baseline and between one - three scans in the premonitory phase. We have previously used a similar design using H_2_^15^O PET with multiple scans in baseline and spontaneous migraine states and found meaningful results with five subjects [22]. Despite the small number in this study, the absence of activation of the NTS and surrounding areas in the no-nausea group and the presence of activation in these areas in the nausea group indicates that these areas are activated when patients experience nausea in the premonitory phase. Also, the results confirmed the a priori hypothesis that these areas would be activated. A drawback of PET studies is the low spatial resolution of the technique, which makes accurate delineation of specific nuclei difficult. Due to the inherent low spatial resolution of PET, and smoothing procedures, we cannot be certain of not missing any activation in smaller structures. Since we have not compared our findings with patients with nausea due to other reasons, we cannot be certain if the results are specific to the premonitory phase of migraine.

## Conclusion

We conclude that occurrence of nausea in the premonitory phase of migraine is associated with activation of the NTS, dorsal motor nucleus of vagus, nucleus ambiguus and the PAG, in the absence of pain, and hence likely represents a primary event. This does not exclude its augmentation by trigeminal nociceptive activation as an additional pathophysiological mechanism. Future research looking into more effective anti-nausea medications for migraine should concentrate on these central sites.

## Competing interests

The authors declare that they have no competing interests.

## Authors’ contributions

FM – study design, study conduction, data analysis, and writing of the manuscript. TS- Study design, study conduction, data analysis, and review of the manuscript. CM – study conduction. PJG - Study design, study conduction, data analysis, and final review of the manuscript. All authors read and approved the final manuscript.

## References

[B1] Headache Classification Committee of the International Headache Society The international classification of headache disorders, 3rd edition (beta version) Cephalalgia 2013 33 629 808 23771276 10.1177/0333102413485658

[B2] Kaube H Keay KA Hoskin KL Bandler R Goadsby PJ Expression of c-*Fos*-like immunoreactivity in the caudal medulla and upper cervical cord following stimulation of the superior sagittal sinus in the cat Brain Res 1993 629 95 102 10.1016/0006-8993(93)90486-7 8287282

[B3] Giffin NJ Ruggiero L Lipton RB Silberstein S Tvedskov JF Olesen J Altman J Goadsby PJ Macrae A Premonitory symptoms in migraine: an electronic diary study Neurology 2003 60 935 940 10.1212/01.WNL.0000052998.58526.A9 12654956

[B4] Maniyar FH Sprenger T Monteith T Schankin C Goadsby PJ Brain activations in the premonitory phase of nitroglycerin triggered migraine attacks Brain 2014 137 232 242 10.1093/brain/awt320 24277718

[B5] Headache Classification Committee of The International Headache Society The international classification of headache disorders (second edition) Cephalalgia 2004 24 Suppl 1 1 160 10.1111/j.1468-2982.2003.00824.x 14979299

[B6] Afridi S Kaube H Goadsby PJ Glyceryl trinitrate triggers premonitory symptoms in migraineurs Pain 2004 110 675 680 10.1016/j.pain.2004.05.007 15288408

[B7] Frackowiak RSJ Friston KJ Functional neuroanatomy of the human brain: positron emission tomography- a new neuroanatomical technique J Anat 1994 184 211 225 8014115 PMC1259983

[B8] Yuan CS Barber WD Area postrema: gastric vagal input from proximal stomach and interactions with nucleus tractus solitarius in cat Brain Res Bull 1993 30 1–2 119 125 8420621 10.1016/0361-9230(93)90047-f

[B9] Boissonade FM Sharkey KA Davison JS Fos expression in ferret dorsal vagal complex after peripheral emetic stimuli Am J Physiol 1994 266 4 Pt 2 R1118 R1126 8184953 10.1152/ajpregu.1994.266.4.R1118

[B10] Shapiro RE Miselis RR The central neural connections of the area postrema of the rat J Comp Neurol 1985 234 3 344 364 10.1002/cne.902340306 3988989

[B11] DeArmond SJ Fusco MM Dewey MM Photographic Atlas – Structure of the Human Brain 1976 2 Oxford University Press, New York

[B12] Nakazawa K Umezaki T Zheng Y Miller AD Behaviors of bulbar respiratory interneurons during fictive swallowing and vomiting Otolaryngol Head Neck Surg 1999 120 412 418 10.1016/S0194-5998(99)70285-8 10064648

[B13] Hyland NP Abrahams TP Fuchs K Burmeister MA Hornby PJ Organization and neurochemistry of vagal preganglionic neurons innervating the lower esophageal sphincter in ferrets J Comp Neurol 2001 430 222 234 10.1002/1096-9861(20010205)430:2<222::AID-CNE1027>3.0.CO;2-Y 11135258

[B14] Subramanian HH Balnave RJ Holstege G The midbrain periaqueductal gray control of respiration J Neurosci 2008 28 12274 12283 10.1523/JNEUROSCI.4168-08.2008 19020021 PMC6671706

[B15] Green AL Hyam JA Williams C Wang S Shlugman D Stein JF Paterson DJ Aziz TZ Intra-operative deep brain stimulation of the periaqueductal grey matter modulates blood pressure and heart rate variability in humans Neuromodulation 2010 13 174 181 10.1111/j.1525-1403.2010.00274.x 21992829

[B16] Ligaya K Horiuchi J McDowall LM Dampney RAL Topographical specificity of regulation of respiratory and renal sympathetic activity by the midbrain dorsolateral periaqueductal gray Am J Physiol Regul Integr Comp Physiol 2010 299 R853 R861 10.1152/ajpregu.00249.2010 20504909

[B17] Horiuchi J McDowall LM Dampney RA Vasomotor and respiratory responses evoked from the dorsolateral periaqueductal grey are mediated by the dorsomedial hypothalamus J Physiol Lond 2009 587 5149 5162 10.1113/jphysiol.2009.179739 19752114 PMC2790255

[B18] Kannan H Yamashita H Connections of neurons in the region of the nucleus tractus solitarius with the hypothalamic paraventricular nucleus: their possible involvement in neural control of the cardiovascular system in rats Brain Res 1985 329 205 212 10.1016/0006-8993(85)90526-8 3978442

[B19] Miller AD Nonaka S Jakus J Brain areas essential or non-essential for emesis Brain Res 1994 647 255 264 10.1016/0006-8993(94)91325-0 7922502

[B20] Robinson BW Mishkin M Alimentary responses to forebrain stimulation in monkeys Exp Brain Res 1968 4 330 366 4976664 10.1007/BF00235700

[B21] Goadsby PJ Hoskin KL The distribution of trigeminovascular afferents in the nonhuman primate brain *Macaca nemestrina*: a c-fos immunocytochemical study J Anat 1997 190 367 375 10.1046/j.1469-7580.1997.19030367.x 9147223 PMC1467617

[B22] Afridi S Giffin NJ Kaube H Friston KJ Ward NS Frackowiak RSJ Goadsby PJ A PET study in spontaneous migraine Arch Neurol 2005 62 1270 1275 10.1001/archneur.62.8.1270 16087768

